# Continuum of care for maternal, newborn, and child health in 17 sub-Saharan African countries

**DOI:** 10.1186/s12913-022-08693-w

**Published:** 2022-11-23

**Authors:** Abdul-Aziz Seidu, Bright Opoku Ahinkorah, Richard Gyan Aboagye, Joshua Okyere, Eugene Budu, Sanni Yaya

**Affiliations:** 1 Centre for Gender and Advocacy, Takoradi Technical University, Takoradi, Ghana; 2 College of Public Health, Medical and Veterinary Sciences, James Cook University, Townsville, Australia; 3 School of Public Health, Faculty of Health, University of Technology Sydney, Sydney, Australia; 4 Department of Family and Community Health, Fred N. Binka School of Public Health, University of Health and Allied Sciences, Hohoe, Ghana; 5 Department of Population and Health, University of Cape Coast, Cape Coast, Ghana; 6 Department of Nursing, Faculty of Allied Health Sciences, College of Health Sciences, Kwame Nkrumah University of Science and Technology, Kumasi, Ghana; 7 School of International Development and Global Studies, University of Ottawa, 120 University Private, K1N 6N5 Ottawa, Canada; 8 The George Institute for Global Health, Imperial College London, London, UK

**Keywords:** Continuum of Care, Maternal health, Child Health, Sub-saharan Africa

## Abstract

**Background:**

Given the intricate relationship between mothers and their children with the continuum of care, it is quintessential for their healthcare and interventions to be enhanced through a continuum of care approach. We examined the factors associated with the continuum of care for maternal, newborn, and child health in sub-Saharan Africa.

**Methods:**

Data for the study were pooled from the Demographic and Health Surveys of 17 countries in sub-Saharan Africa. We extracted the data from the women’s files in all 17 countries. We included 15,359 married and cohabiting women with the most recent children aged 12–23 months at the time of the survey in the study. Multivariable multilevel logistic regression analysis was performed to examine the factors associated with continuum of care. Results were presented as adjusted odds ratios (aORs) with their 95% confidence interval.

**Results:**

Women whose partners had secondary or higher level of education [aOR = 1.52; 95%CI = 1.07, 2.16], those with health insurance [aOR = 1.64; 95%CI = 1.18, 2.30], those who decide alone on their healthcare [aOR = 1.38; 95%CI = 1.01, 1.89], those with joint healthcare decision [aOR = 1.33; 95%CI = 1.02, 1.74], those exposed to radio [aOR = 1.38; 95%CI = 1.06, 1.79], those who started antenatal care early [aOR = 1.88; 95%CI = 1.50, 2.36] and those in southern Africa [aOR = 7.02; 95%CI = 3.23, 15.27] had higher odds of completing the continuum of care.

**Conclusion:**

We found that only 3.4% of women across the 17 sub-Saharan African countries included in this study completed all of the 11 maternal, newborn, and child health care interventions along the continuum of care. The factors that are associated with the maternal, newborn, and child health continuum of care include women’s autonomy in decision-making, partners’ level of education, health insurance coverage, early initiation of antenatal care, exposure to radio, and residing in Southern Africa. Problem with the distance to the facility lowered women’s odds of completing the continuum of care. Governments and departments of health services across sub-Saharan African countries must leverage the radio to disseminate critical messages about the need to complete the continuum of care. Much commitment must be made toward advancing the autonomy of women. Health insurance coverage would have to be extended to reach all women to facilitate the completion of the continuum of care.

## Background

Reducing maternal and child mortality remains an important indicator of the development and overall health status of communities. Global estimates indicate that between 2000 and 2017, the maternal mortality ratio (MMR) worldwide reduced by nearly 38%, with a 2.9% decline on average per annum [1]. Notwithstanding this improvement in MMR, there must be an annual average decline of 6.4% before countries can attain the Sustainable Development Goal (SDG) target 3.1, that is, reduce MMR to less than 70 deaths per 100,000 live births [2, 3]. Relatedly, evidence shows that the under-five mortality rate has declined across the globe from 75 deaths per 1,000 live births in 2000 to 38 deaths per 1,000 live births in 2019 [4]. Yet, the under-five mortality rate remains unacceptably high in comparison to the target of 25 deaths per 1000 live births as articulated by the SDG target 3.2. Hence, this underscores the need to find evidence-based strategies to promote a further reduction in maternal and child mortalities.

Sub-Saharan Africa (SSA) records some of the highest rates of maternal and child mortality. For instance, it is known from the literature that SSA accounts for almost 90% of the global burden of maternal mortality [5]. This high burden of maternal and child mortality in SSA has been linked to several preventable factors including low antenatal care (ANC) utilisation as well as low uptake of postnatal care (PNC), skilled attendant delivery, and health facility delivery [3, 6]. Moreover, evidence suggests that easy accessibility to maternal and child healthcare is associated with significantly lower odds of child and maternal mortalities [7, 8]. The WHO has also indicated that affordability, availability, and quality of maternal and child healthcare services are critical to reducing the risk of both maternal and child deaths [8]. Therefore, providing maternal and child healthcare (MCH) through a continuum of care (CoC) could significantly reduce maternal and child mortality worldwide, particularly in SSA.

CoC is an approach that ensures continuous care throughout the life cycle. In the context of MCH, CoC encapsulates healthcare provided from the period of pregnancy, through to childbirth, postnatal period, infancy, and the childhood period [3, 9]. CoC is an important approach for viewing both mother and child as a collective rather than as individual entities. Notwithstanding, MCH policies and interventions particularly in SSA have often viewed mothers and their children as separate entities rather than as a collective, hence creating substantial gaps in the already existing MCH interventions [3, 10]. Given the intricate relationship between mothers and their children with the continuum, it is quintessential for their healthcare and interventions to be enhanced through a CoC approach. CoC is a recommended approach for discussing MCH because it is made up of the dimension of time and place [11]. The time highlights the continuity of care over time at different stages of pregnancy, childbirth, and postpartum, whereas the place dimension links various levels of services provided at home, communities, and health facilities [11]. This provides a holistic framework to understand MCH.

There is the preponderance of evidence in SSA that have examined factors associated with individual components of the CoC. For instance, Aboagye et al. [6] assessed timely ANC attendance in SSA. Other studies conducted in SSA have also examined the factors associated with skilled birth attendance [12], home deliveries [13], PNC utilisation [14], breastfeeding [15], and vaccination [16]. However, there is a dearth of empirical evidence on the factors associated with the CoC for maternal, newborn, and child health (MNCH) in SSA. To the best of our knowledge, only three studies have been conducted on the factors associated with the CoC in SSA using nationally representative data [3, 17, 18]. However, two of these studies were conducted only in the Gambia [3] and Uganda [17], as such the findings may not necessarily be extrapolated and generalized to the entire sub-Saharan African region. The third study which actually captured 12 sub-Saharan African countries also used only six indicators for measuring MNCH CoC [18], whereas our study uses 11 indicators. Moreover, the previous studies relied on data from 2010 to 2018. However, our study uses much recent data from 2015 to 2020. To bridge this literature gap, we examined the factors associated with the CoC for MNCH in SSA. The findings are critical and could provide a basis for policy formulation and implememtation in improving MNCH through CoC.

## Methods

### Data source and study design

We performed a secondary analysis of data pooled from the Demographic and Health Surveys (DHSs) of 17 countries in SSA. We extracted the data from the women’s file (IR Recode) in all 17 countries. The “IR Recode” is the individual recode file that contains the dataset for women of reproductive age. We based the inclusion criteria on the following; (i) countries with recent datasets conducted from 2015 to 2020 and (ii) countries with complete cases on the variables of interest. Hence, we included only 17 countries (Table 1). DHS is a nationally comparative survey conducted in over 90 low-and middle-income countries globally [19]. The DHS program is funded by the United State Agency for International Development (USAID). Other source of funding comes from the US Centers for Disease Control and Prevention (CDC), the United Nations Population Fund (UNFPA), the United Nations Children’s Fund (UNICEF), the Irish Embassy (Irish Aid), the United Nations Development Programme (UNDP), the World Health Organization (WHO), UN Women, and the Global Alliance for Vaccine and Immunization (GAVI) [19]. Inner City Fund provides technical assistance through the DHS Program, a USAID-funded effort that provides support and technical assistance in the implementation of DHSs in numerous countries.

**Table 1:** Description of the study sample and prevalence of continuum of care in sub-Saharan Africa

Country	Year of survey	Weighted N	Weighted %	Continuum of care
1. Angola	2015-16	641	4.2	1.2 [0.4–3.6]
2. Benin	2017-18	1712	11.2	2.5 [1.8–3.6]
3. Burundi	2016-17	1149	7.5	2.4 [1.5–3.8]
4. Cameroon	2018	651	4.2	0.9 [0.4–2.0]
5. Ethiopia	2016	1104	7.2	3.4 [2.1–5.4]
6. Gambia	2019-20	566	3.7	3.8 [2.3–6.2]
7. Guinea	2018	513	3.3	0.6 [0.2–2.5]
8. Liberia	2019-20	228	1.5	5.3 [3.3–8.3]
9. Mali	2018	1293	8.4	3.2 [2.3–4.5]
10. Malawi	2015-16	838	5.5	8.8 [6.4–12.1]
11. Nigeria	2018	1737	11.3	1.1 [0.7–1.8]
12. Rwanda	2019-20	613	4.0	8.9 [6.9–11.5]
13. Sierra Leone	2019	650	4.2	6.0 [4.0–8.7]
14. Tanzania	2015-16	1572	10.2	1.1 [0.7–2.0]
15. Uganda	2016	683	4.4	4.9 [3.3–7.2]
16. South Africa	2016	103	0.7	7.7 [2.8–19.1]
17. Zambia	2018	1306	8.5	5.2 [4.0–6.7]
**All countries**	**2015–2020**	**15,359**	**100.0**	**3.4 [3.0–3.7]**

The DHS employs a structured questionnaires were used to collect data from the respondents. Trained research assistants conducted the face-to-face interview. The face-to-face interviews were conducted in the respective country’s official language. A two-stage cluster sampling approach was used to choose respondents for the DHS survey. The initial phase was selecting sample locations (clusters) comprised of enumeration areas (EAs). The second stage was a systematic sample of houses. A household listing operation was done in each of the designated EAs, and households to be included in the survey were selected at random from the list. The detailed sampling technique used in the DHS has been highlighted in the literature [20]. The sample size included 15,359 married and cohabiting women with the most recent children aged 12–23 months at the time of the survey in the study. In addition, the sample consisted of only women with complete observations on the variables of interest in this study (See Table 1). The datasets can be accessed at https://dhsprogram.com/data/available-datasets.cfm.

### Variables

#### Outcome variable

CoC was the outcome variable in this study. Presently, no studies have stipulated the measurement of CoC using a standardized approach. Hence, we calculated the CoC using the composite coverage metrics called the co-coverage index (CCI) from the literature [3, 7, 21, 22] as well as the WHO recommendation for MNCH care [20]. In our study, we calculated CoC from eleven (11) essential MNCH interventions. They include ANC, neonatal tetanus toxoid injection, health facility-based delivery, skilled birth delivery, PNC within the first two days after birth, Bacille de Calmette et Guérin vaccination (BCG), measles vaccination, diphtheria–pertussis–tetanus vaccine (DPT), poliomyelitis vaccine (Polio), age-appropriate breastfeeding, and current use of modern contraceptive use. We coded all the women and their children aged 12–23 months who received the 11 interventions as “1”, indicating that they had CoC. The remaining women and children were categorized as having incomplete CoC and were recoded as “0”. A detailed explanation of the variables utilized in estimating the Coc and its coding process has been highlighted in the literature [3, 11, 23].

#### Explanatory variables

We considered twenty explanatory variables in this study. These variables were selected based on their significant association with MNCH care from the literature [3, 22, 24]. We grouped the variables into individual-level factors consisting of the mother and child characteristics and household or community-level factors. The individual-level variables consisted of age and educational level of the women and their partners, current working status, marital status, health insurance coverage, wanted the last child when pregnant, person who usually decides on respondents’ health care, exposure to watching television, exposure to listening to radio, exposure to reading newspaper or magazine, timely ANC visits, getting medical help for self: distance to health facility, sex of child, birth order, and birth size. Wealth index, sex of household head, place of residence, and geographical sub-regions were the household or community level variables. The categorization of each variable is shown in Table 2.

**Table 2:** Distribution of continuum of care across the explanatory variables

Variable	Weighted N (%)	Continuum of care % [95% CI]	P-value	cOR [95% CI]
**Women’s age (years)**			0.212	
15–24	4,395 (28.6)	3.4 [2.8–4.1]		1.0
25–34	7,562 (49.2)	3.6 [3.1–4.1]		1.06 [0.83–1.35]
35 and above	3,402 (22.2)	2.8 [2.2–3.6]		0.82 [0.60–1.12]
**Mothers’ level of education**			< 0.001	
No education	5609 (36.5)	1.9 [1.5–2.4]		1.0
Primary	5666 (36.9)	3.5 [3.0–4.1]		1.87*** [1.42–2.47]
Secondary or higher	4084 (26.6)	5.1 [4.3–6.0]		2.74*** [2.06–3.64]
**Current working status**			0.695	
No	4852 (31.6)	3.2 [2.7–3.9]		1.0
Yes	10,507 (68.4)	3.4 [3.0–3.9]		1.05 [0.83–1.32]
**Marital status**			0.411	
Married	12,624 (82.2)	3.4 [3.0–3.8]		1.0
Cohabiting	2735 (17.8)	3.0 [2.4–3.9]		0.89 [0.67–1.18]
**Partner’s level of education**			< 0.001	
No education	4796 (31.2)	1.9 [1.5–2.4]		1.0
Primary	5270 (34.3)	3.1 [2.6–3.7]		1.67** [1.24–2.27]
Secondary or higher	5293 (34.5)	4.9 [4.3–5.7]		2.74*** [2.05–3.66]
**Health insurance coverage**			< 0.001	
No	14,146 (92.1)	3.1 [2.8–3.5]		1.0
Yes	1213 (7.9)	6.2 [4.9–7.9]		2.08*** [1.57–2.75]
**Wanted last child when pregnant**			0.492	
Wanted then	11,422 (74.4)	3.5 [3.1–3.9]		1.0
Wanted later	3168 (20.6)	3.0 [2.3–3.8]		0.86 [0.65–1.13]
Wanted no more	769 (5.0)	3.1 [2.0–4.8]		0.89 [0.56–1.43]
**Person who usually decides on respondents health care**			< 0.001	
Partner alone/someone/others	6222 (40.5)	2.4 [1.9–2.8]		1.0
Respondent/partner	6789 (44.2)	3.8 [3.3–4.5]		1.66*** [1.30–2.12]
Respondent alone	2348 (15.3)	4.5 [3.6–5.7]		1.97*** [1.46–2.66]
**Exposed to watching television**			0.010	
No	9293 (60.5)	3.0 [2.6–3.4]		1.0
Yes	6066 (39.5)	3.9 [3.3–4.6]		1.32* [1.07–1.64]
**Exposed to listening to radio**			< 0.001	
No	6502 (42.3)	2.5 [2.1–3.0]		1.0
Yes	8857 (57.7)	4.0 [3.5–4.5]		1.62*** [1.29–2.03]
**Exposed to reading newspaper or magazine**			< 0.001	
No	13,136 (85.5)	2.9 [2.6–3.3]		1.0
Yes	2223 (14.5)	5.8 [4.6–7.2]		2.03*** [1.55–2.65]
**Timely antenatal care visits**			< 0.001	
Late antenatal care visit	9311 (60.6)	2.4 [2.1–2.9]		1.0
Early antenatal care visit	6048 (39.4)	4.7 [4.1–5.4]		2.00*** [1.61–2.48]
**Getting medical help for self: distance to health facility**			< 0.001	
Not a big problem	9658 (62.9)	3.9[3.4–4.4]		1.0
Big problem	5701 (37.1)	2.4 [2.0–3.0]		0.61*** [0.48–0.78]
**Sex of child**			0.645	
Male	7875 (51.3)	3.3 [2.8–3.8]		1.0
Female	7484 (48.7)	3.4 [2.9–4.0]		1.05 [0.85–1.31]
**Birth order**			0.001	
1	2775 (18.1)	4.4 [3.6–5.4]		1.0
2–3	5656 (36.8)	3.7 [3.1–4.3]		0.82 [0.62–1.08]
4 or more	6928 (45.1)	2.6 [2.2–3.1]		0.59*** [0.45–0.77]
**Birth size**			0.172	
Large	4889 (31.8)	3.8 [3.2–4.5]		1.0
Average	8325 (54.2)	3.1 [2.7–3.6]		0.80 [0.64–1.02]
Small	2145 (14.0)	3.2 [2.4–4.2]		0.83 [0.60–1.15]
**Wealth index**			< 0.001	
Poorest	3130 (20.4)	2.6 [2.0–3.3]		1.0
Poorer	3212 (20.9)	2.4 [1.8–3.1]		0.90 [0.63–1.30]
Middle	3258 (21.2)	3.1 [2.5–3.8]		1.18 [0.85–1.63]
Richer	2993 (19.5)	3.1 [2.4–4.0]		1.20 [0.83–1.73]
Richest	2766 (18.0)	5.9 [4.8–7.2]		2.34*** [1.69–3.25]
**Sex of household head**			0.767	
Male	13,329 (86.8)	3.4 [3.0–3.8]		1.0
Female	2030 (13.2)	3.2 [2.5–4.2]		0.96 [0.71–1.28]
**Place of residence**			0.003	
Urban	4907 (31.9)	4.2 [3.5–5.0]		1.0
Rural	10,452 (68.1)	3.0 [2.6–3.4]		0.70** [0.56–0.88]
**Geographical subregions**			< 0.001	
Central	1292 (8.4)	1.1 [0.5–2.2]		1.0
Western	6699 (43.6)	2.7 [2.3–3.2]		2.59* [1.22–5.48]
Eastern	5121 (33.4)	3.3 [2.8–4.0]		3.24** [1.53–6.86]
Southern	2247 (14.6)	6.7 [5.4–8.2]		6.72*** [3.14–14.38]

*P-values were generated from chi-square test; cOR = crude odds ratios; CI = Confidence Interval; ^*^p < 0.05, ^**^p < 0.01, ^***^p < 0.001; 1 = Reference category

### Statistical analyses

All statistical analyses were performed using Stata software, version 16.0. We first presented the results of the MNCH CoC using percentages with their respective 95% confidence interval. Subsequently, the results of each component of the CoC were summarized and presented in a tabular form using percentages (Table 3). Cross-tabulation test was adopted to determine the distribution of CoC across the explanatory variables. We used a univariable binary logistic regression test to examine the independent association between the explanatory variables and CoC. All the statistically significant variables were moved to the multivariable multilevel binary logistic regression analysis. In performing the multilevel regression analysis, four models were used. Model O, which was an empty model with no explanatory variables, demonstrated the variance in CoC that could be attributed to the primary sample units (PSUs). Model I only included the individual-level variables, whereas Model II included it as well as household/community-level variables. The final model (Model III) took into account all explanatory variables. The results of the regression analysis were tabulated, yielding adjusted odds ratios (aOR) with 95% confidence intervals (CIs). For all variables, a p-value of < 0.05 was considered statistically significant. Furthermore, each of the four models incorporated both fixed and random effects. Fixed effects denoted the measure of variation in Coc variables based on PSUs (ICC), whereas random-effects denoted the measure of variation in CoC based on the explanatory variables. Akaike’s Information Criterion (AIC) was used to assess model fitness, or how well different models match the data. Stata’s “melogit” function was used to run the multilevel regression models. To correct for disproportionate sampling and non-response, the “svyset” command was employed to accommodate the intricate nature of DHS data. We followed the Strengthening Reporting of Observational Studies in Epidemiology (STROBE) reporting requirements when writing this paper [25].

**Table 3:** Maternal, newborn, and child health and continuum of care

Maternal, newborn, and child health and continuum of care indicators	Frequency (N)	Percentage (%)
Antenatal care (4 or more visits)	9728	63.3
Neonatal tetanus protection	8180	53.3
Facility-based delivery	11,549	75.2
Skilled birth delivery	10,854	70.7
Postnatal care within the first 2days after birth	7657	49.9
BCG	14,101	91.8
DPT	12,154	79.1
Polio	10,379	67.6
Measles	11,943	77.7
Age appropriate breastfeeding	10,967	71.4
Current use of modern contraceptive	5,139	33.5

### Ethical consideration

In this study, we did not seek ethical approval the conduct this study because the data is publicly available for use. Before obtaining and using it for the study, permission was obtained from the MEASURE DHS and approval was granted. Prior to the survey’s start, ethical permission was acquired, and all ethical requirements governing the use of human subjects were properly followed. The detailed ethical guidelines can be found at http://goo.gl/ny8T6X.

## Results

Table 1 shows the description of the study sample and the prevalence of CoC in MNCH services utilisation among women. It was found that the average completion of the CoC for all the 17 countries surveyed was 3.4% and this ranged from 0.6% in Guinea to 8.9% in Rwanda. Table 3 shows the results on MNCH and CoC.

### Maternal, newborn, and child health and continuum of care in the 17 sub-Saharan African countries

Table 3 shows the results on MNCH coverage and CoC among the married and cohabiting women. The results showed that 63.3% of the women attended ANC four or more times, 70.7% had skilled birth delivery, and 49.9% had PNC with the first 2days after delivery. Among the four vaccines included in the MNCH, majority (91.8%) of the reported that their child aged 12–23 months has completed the recommended dose for BCG, followed by DPT (79.1%), and measles (77.7%). Additionally, 71.4% of the mothers breasfed their child with the age-apprpriate breastfeeding and 33.5% used modern contraceptives.

### Distribution of continuum of care across the explanatory variables

Table 2 presents the findings on the distribution of CoC across the explanatory variables. Women aged 25–34 had 3.6% ofCoC, women with a secondary or higher level of education (5.1%), women with health insurance (6.2%), women who decides alone on healthcare (4.5%), exposed to TV (3.9%), radio (4.0%) and newspaper (5.8%) had higher proportions of CoC. It was also found that women who made early antenatal care visits (4.7%), those who had first birth order (4.4%), those in the richest wealth quintile (5.9%), those in urban (4.2%) and those in southern Africa (6.7%) had highest proportions of CoC. The chi-square analysis showed statistically significant differences in the proportions across all the variables except age of the mother, current working status, marital status, wanted the last child when pregnant, sex of household head, birth size, and sex of the child at p < 0.05. This was also confirmed by the crude regression analysis.

### Mixed effect analysis of factors associated with continuum of care in sub-Saharan Africa

#### Fixed effects results

Table 4 shows the results on the mixed effect analysis of factors associated with CoC in sub-Saharan Africa. It was found that women whose partners had secondary or higher level of education [aOR = 1.52; 95%CI = 1.07, 2.16], those with health insurance [aOR = 1.64; 95%CI = 1.18, 2.30], those who decide alone on their healthcare [aOR = 1.38;95%CI = 1.01, 1.89], those with joint healthcare decision [aOR = 1.33; 95%CI = 1.02, 1.74], those exposed to radio [aOR = 1.38; 95%CI = 1.06, 1.79], those who started ANC early [aOR = 1.88; 95%CI = 1.50, 2.36] and those in southern Africa [aOR = 7.02; 95%CI = 3.23, 15.27] had higher odds of completing the CoC compared with those whose partners had no formal education, those without health insurance, those whose partner alone/someone/others decides on their healthcare, not exposed to radio, late ANC visits and those in Central Africa. However, those who had a big problem with distance to health facility **[**aOR = 0.73; 95%CI = 0.57, 0.95] had lower odds of completing the CoC compared with those who had no problem with distance to health facility.

**Table 4:** Mixed effect analysis of factors associated with CoC in sub-Saharan Africa

Variables	Model O	Model I aOR [95% CI]	Model II aOR [95% CI]	Model III aOR [95% CI]
**Fixed effect results**				
**Mothers’ level of education**				
No education		1.00		1.00
Primary		1.35^*^ [1.00, 1.82]		1.20 [0.87, 1.65]
Secondary or higher		1.39 [0.98, 1.97]		1.20 [0.83, 1.74]
**Partner’s level of education**				
No education		1.00		1.00
Primary		1.26 [0.92, 1.73]		1.18 [0.86, 1.64]
Secondary or higher		1.78^***^ [1.28, 2.48]		1.52^*^ [1.07, 2.16]
**Health insurance coverage**				
No		1.00		1.00
Yes		1.55^**^ [1.15, 2.10]		1.64^**^ [1.18, 2.30]
**Person who usually decides on respondents health care**				
Partner alone/someone/others		1.00		1.00
Respondent/partner		1.39^*^ [1.07, 1.80]		1.33^*^ [1.02, 1.74]
Respondent alone		1.65^**^ [1.21, 2.25]		1.38^*^ [1.01, 1.89]
**Exposed to watching television**				
No		1.00		1.00
Yes		0.79 [0.60, 1.05]		0.84 [0.61, 1.14]
**Exposed to listening to radio**				
No		1.00		1.00
Yes		1.36^*^ [1.05, 1.77]		1.38^*^[1.06, 1.79]
**Exposed to reading newspaper or magazine**				
No		1.00		1.00
Yes		1.34 [0.99, 1.83]		1.31 [0.95, 1.79]
**Timely antenatal care visits**				
Late antenatal care visit		1.00		1.00
Early antenatal care visit		1.82^***^ [1.46, 2.28]		1.88^***^ [1.50, 2.36]
**Getting medical help for self: distance to health facility**				
Not a big problem		1.00		1
Big problem		0.70^**^ [0.55, 0.91]		0.73^*^ [0.57, 0.95]
**Birth order**				
1		1.00		1.00
2–3		0.88 [0.66, 1.18]		0.87 [0.65, 1.18]
4 or more		0.79 [0.59, 1.06]		0.80 [0.59, 1.08]
**Wealth index**				
Poorest			1.00	1.00
Poorer			0.93 [0.63, 1.37]	0.85 [0.58, 1.24]
Middle			1.29 [0.90, 1.84]	1.09 [0.76, 1.55]
Richer			1.31 [0.88, 1.95]	0.97 [0.65, 1.45]
Richest			2.51^***^ [1.73, 3.66]	1.44 [0.94, 2.21]
**Place of residence**				
Urban			1.00	1.00
Rural			0.96 [0.73, 1.28]	1.06 [0.78, 1.44]
**Geographical subregions**				
Central			1.00	1.00
Western			2.64^*^ [1.24, 5.64]	3.15^**^ [1.45, 6.85]
Eastern			3.39^**^ [1.57, 7.30]	3.11^**^ [1.41, 6.85]
Southern			7.50^***^ [3.44, 16.34]	7.02^***^ [3.23, 15.27]
**Random effect results**				
PSU variance (95% CI)	0.526 [0.334–0.829]	0.509 [0.322–0.806]	0.488 [0.310 − 0.768]	0.507 [0.322–0.799]
ICC	0.138	0.134	0.129	0.133
Wald chi-square	Reference	141.41 (< 0.001)	111.66 (< 0.001)	209.84 (< 0.001)
**Model fitness**				
Log-likelihood	-2218.3041	-2115.7183	-2136.8255	-2071.6726
AIC	4440.608	4263.437	4293.651	4191.345
BIC	4455.887	4385.668	4370.045	4374.692
N	15,359	15,359	15,359	15,359
Number of clusters	1188	1188	1188	1188

aOR = Adjusted Odds Ratios; CI = Confidence Interval; ^*^p < 0.05, ^**^p < 0.01, ^***^p < 0.001; 1 = Reference category; PSU = Primary Sampling Unit; ICC = Intra-Class Correlation; AIC = Akaike’s Information Criterion; PSU: Primary Sampling Unit

#### Random effects results

The results of the random effects are also presented in Table 4. It was shown that the ICC value for the null model was 14% which shows that about 14% of the variation in the completion of the CoC is attributed to variation between clusters. This variation reduced slightly to 13% in the individual level as well as all the other models. Model III which is the complete model with individual, household and community level variables had a lower Akaike Information Criterion (4191.345) compared to the other models affirming the goodness of fit.

## Discussion

We examined the factors associated with the CoC for MNCH in SSA. Our findings show that only 3.3% of women in SSA completed 11 MNCH care interventions along with the CoC. The prevalence of completing MNCH care interventions in the present study is relatively greater when compared to the 1.8% completion of 11 MNCH care interventions that was reported in a related study in the Gambia [3]. Additionally, our findings reveal that the lowest completion coverage along the CoC was in the area of current use of modern contraceptives (33.5%). This is synonymous with Oh et al.’s [3] findings. Possibly, the low coverage in terms of modern contraceptive use could be attributable to women’s low autonomy in making healthcare decisions [26, 27]. Another plausible explanation could be due to the timing for data collection under the DHS. According to Oh et al. [3], the DHS collected data on contraceptive prevalence after delivery, and thus, explains the low coverage of current modern contraceptive use along the CoC. Evidence suggests that lactation provide some sort of protection against pregnancy [28]. Therefore, it is possible that the low coverage in the use of modern contraceptives could be a reflection of high rates of breastfeeding in SSA.

Women’s autonomy in making healthcare decision emerged significantly associated with the CoC. We found that women who autonomously made decisions regarding their healthcare were 1.38 times more likely to complete the CoC as compared to those who depended on their partners to make healthcare decisions. The result is corroborated by the findings of earlier studies conducted in the Gambia [3], Pakistan [11] as well as in South Asia and SSA [29]. Probably, we may explain this result from the perspective that women’s health decision-making autonomy precludes men or their male partners from exerting their dominance and influence over women’s decision to go for ANC, PNC, use modern contraceptives, seek vaccination for children, and other MNCH care intervention along with the CoC.

Analogous to the issue of autonomy, we found that women whose partners had secondary or higher level of education were 1.52 times more likely to complete MNCH care interventions along the CoC as compared to those whose partners had only primary or no formal education. This finding is consistent with that of Oh et al. [3] and Iqbal et al. [11]. We postulate that partners with a higher level of formal education are most likely to respect women’s decision concerning their healthcare-seeking throughout the CoC. Through high level of formal education, male partners become exposed to the importance of ensuring that their partners complete the CoC. Hence, they are likely to become supportive and encourage their wives to complete the CoC. Closely knitted to this finding is our result that shows that SSA women who are exposed to the radio have greater odds of completing MNCH care interventions along with the CoC. Similar findings have been reported in Gambia [3] and Ethiopia [30]. Thissuggests that the delivery of well-crafted MNCH messages through the radio is likely to yield much positive impact on women’s completion of MNCH care interventions along with the CoC than when delivered through the television or other media platforms.

Consistent with the findings of a related study conducted in Mexico [31], we found a statistically significant association between health insurance coverage and MNCH CoC. Women who had health insurance coverage were 1.64 times more likely to complete all 11 MNCH care interventions along with the CoC as compared to their colleagues who had no health insurance coverage. Often, women miss out on MNCH care intervention due to out-of-pocket-payment that usually lead to catastrophic health expenditure [6]. As such, health insurance becomes the intervention that allows them to go through the CoC while minimizing or completely avoiding out-of-pocket payments. Relatedly, women who had a big problem with distance to health facility were less likely to complete the CoC compared to those who had no problem with distance to health facility. This finding mirrors the results of Oh et al. [3]. Long distance suggests that women would have to secure money to offset their transportation cost [32]. This tends to dissuade many women, particularly rural dwelling women and those who are not economically active from completing the CoC.

Early initiation had positive association with the CoC. Women who started ANC early had greater odds of completing all of the 11 MNCH care interventions along the CoC. Our finding aligns with Oh et al.’s study [3]. Perhaps, we could explain this finding from the point that, during ANC sessions, women are taken through a series of health education. It is during ANC sessions that different cadres of healthcare providers explain the need for women to complete the continuum of care, and how completing the CoC can help in preventing certain pregnancy-related complicated and avoidable deaths [17]. Also, women are more likely to develop a good rapport with healthcare providers when they initiate ANC early; this relationship building goes a long way to influence women’s decision to proceed with skilled birth delivery, return for PNC, and vaccination for the child among others in the CoC [33]. Our study also shows that women in southern Africa were 7.02 times more likely to complete the 11 MNCH care interventions along with the CoC. We are unable to provide a plausible explanation for this result. As such, dedicated empirical investigations could be done in Southern Africa to ascertain the reasons for this finding.

### Policy implications

The results suggest that there is a need to implement policies and programs that will facilitate women’s completion of all MNCH care interventions along the CoC. The established association between women’s autonomy in healthcare decision and the CoC emphasizes the need for more commitment at the regional and national level toward improving women’s autonomy. This could be in the form of providing skills training and human capital development to women. Such actions would translate to self-reliance and ultimately autonomy in decision-making. Again, the findings from this study bring to the fore the intricate role of male partners in promoting the completion of the CoC. Advocacies, public education, policies, and other programs that educate women on the need to have a completed CoC must consciously make room for male involvement sessions to raise partners’ awareness and draw their minds to being supportive in women’s quest to complete the CoC. The problem with distance to health facility implies that SSA countries must initiate decentralized healthcare provision through modules like community-health-based centers.

### Strength and limitations

The use of the DHS limits the kind of analysis and inferences that can be made from our findings. Due to the cross-sectional nature of the DHS, we are unable to establish causality between the factors associated with the CoC. Also, as a study from a secondary dataset, we are unable to assess the association between other residual variables such as the influence of culture, among others. The data was self-reported. As such, there is the possibility of social desirability bias and recall bias. Notwithstanding these limitations, we have a strong conviction that the use of a nationally representative dataset ensures that our findings are generalizable to the larger population. We applied robust analysis in this study which adds to the validity of the study findings. Also, this study is arguably the first to examine the factors associated with MNCH CoC from the regional perspective.

## Conclusion

We found that only 3.4% of women across the 17 SSA countries included in this study completed all of the 11 MNCH care interventions along with the CoC. The factors that are associated with the MNCH CoC include women’s autonomy in decision making, partners’ level of education, health insurance coverage, early initiation of ANC, exposure to radio and residing in Southern Africa. Problem with the distance to the facility lowered women’s odds of completing the CoC. Governments and departments of health services across SSA countries must leverage on the radio to disseminate critical messages about the need to complete the CoC. Much commitment must be made toward advancing the autonomy of women. Health insurance coverage would have to be extended to reach all women to facilitate the completion of the CoC.

## Data Availability

Data for this study were sourced from Demographic and Health surveys (DHS) and available here: http://dhsprogram.com/data/available-datasets.cfm.
